# Ultrasensitive specific sensor based on all-dielectric metasurfaces in the terahertz range

**DOI:** 10.1039/d0ra06463g

**Published:** 2020-09-07

**Authors:** Yajun Zhong, Lianghui Du, Qiao Liu, Liguo Zhu, Kun Meng, Yi Zou, Bin Zhang

**Affiliations:** College of Electronics and Information Engineering, Sichuan University Chengdu 610065 China zhangbinff@sohu.com; Institute of Fluid Physics, China Academy of Engineering Physics Mianyang 621900 China Lianghui_Du@163.com; Microsystem and Terahertz Research Center, China Academy of Engineering Physics Mianyang 621900 China

## Abstract

An ultrasensitive specific sensor based on all-dielectric metasurfaces in the terahertz range was proposed. The designed metasurfaces consist of multi pairs of tilted silicon bars on a SiO_2_ substrate with a high-*Q* Fano resonance feature. The peak of this high *Q* Fano resonance can form a wide reflection spectrum band by scanning the angle of the incident THz waves. Utilizing this angle-scanning strategy, we designed a metasurface sensor and its reflection spectrum band can cover the absorption peak of tyrosine and santonin. By depositing different thicknesses of tyrosine and santonin on the sensor, we have successfully identified them with a detection limit of 6.7 μg cm^−2^ and 59.35 μg cm^−2^, respectively. The performance of the sensor with high sensitivity has been analyzed in detail, showing an exciting prospect for identification of ‘fingerprint’ spectra in the terahertz region.

## Abstract 2

An ultrasensitive specific sensor based on all-dielectric metasurfaces in the terahertz range was proposed.

## Graphical abstract

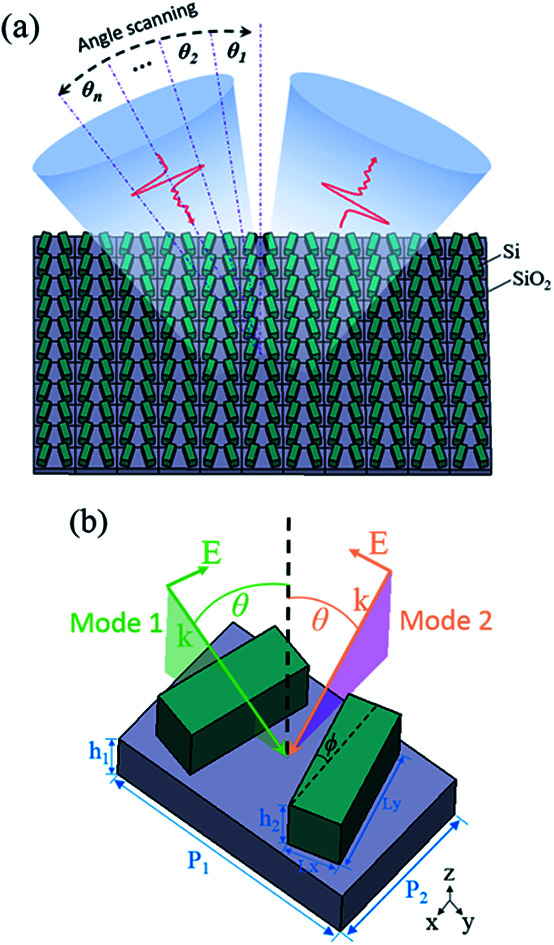

## 1. Introduction

As one of the important applications for terahertz (THz) technologies, sensing has been investigated extensively in recent years for its ability to achieve non-destructive detection, gas sensing, and biomedicine identification.^1–3^ Terahertz time-domain spectroscopy (THz-TDS) has been demonstrated to be a powerful tool for characterization and identification of chemicals and biological materials because the rotational and vibrational modes of bio/chem-molecules lies in the THz region.^4,5^ However, the poor sensitivities (typical amount of ∼100 mg) of the THz-TDS system restrict its applications in the field of biochemistry, where trace amounts of analytes need to be detected effectively and accurately.^6^

In order to detect trace amounts of analytes in the THz range, metal-based metasurfaces with large local electromagnetic field enhancement are firstly put forward to improve the interaction between THz waves and analytes.^7^ Fano type resonances are then introduced into the metallic metasurfaces to further improve the *Q* factor, which usually means stronger electromagnetic field confinement and higher sensitivity.^8,9^ A maximum sensitivity of >1 THz/RIU have been achieved at the Fano mode in the THz band.^10^ However, the *Q*-factors of metal-based resonant systems are limited to <∼10 due to inevitable non-radiative loss.^11^ Recently, researchers used the all-dielectric THz metasurfaces to overcome the ohmic loss of metallic material and achieved high *Q* values of up to 500 and FOM of 12.7.^12,13^ However, metasurfaces sensors with high sensitivity are mainly focus on the refractive index sensing without the ability to identify analytes.^14–16^ Meanwhile, the narrow linewidth of resonance accompanying with high-*Q* sensor makes it difficult to cover the analyte's ‘fingerprint’ spectrum.^17^ And once fabricated, the resonance features cannot be changed in both metallic and dielectric metasurfaces. In order to achieve specific sensing, matematerials with additional phase-transition materials or graphene can be added to the metasurface to achieve the dynamic control of resonant frequency.^18–20^ With this resonant frequency scanning method, the resonance frequency of high-*Q* sensor can cover the absorption peaks of analytes making it able to identify analytes. The specific sensing of benzoic acid with detection limit smaller than 6.35 μg cm^−2^ have been achieved based on graphene micro-ribbon array structure.^21^ Unfortunately, these methods require additional photoelectric equipments, which complicates the system's setup. Therefore, in order to promote the development of THz specific sensing with high sensitivity, it is still of great significance and challenge to design metasurface sensors that can identify the fingerprint spectrum of the analytes in the THz region.

In this paper, an ultrasensitive specific sensor based on all-dielectric metasurfaces with frequency scanning in the THz range has been designed with the help of the newly developed angle-multiplexed technology in the infrared band.^22^ The unit structure of the metasurface consists of pairs of tilted silicon bars placed on a SiO_2_ substrate, which can excite resonance with high *Q* factor based on quasi bound states in the continuum. When the incident angles of the THz waves are scanned in two different polarization modes, the resonance frequency will be red or blue shifted, which eventually forms a wide reflection spectrum. By analyzing the refractive index sensing characteristics of the metasurface, it reveals that the sensitivities is 77 GHz/RIU and figure of merits (FOM) is 11.1. In addition, the absorption peaks of the tyrosine and santonin can be recognized by the metasurface sensor, and the detection limit of the tyrosine and the santonin are 6.7 μg cm^−2^ and 59.35 μg cm^−2^, respectively, which demonstrates a new way to fingerprint spectrum recognition with high sensitivity and provides an effective and feasible technical support to the target analytes detection.

## 2. Structure and resonance properties

**Fig. 1:**
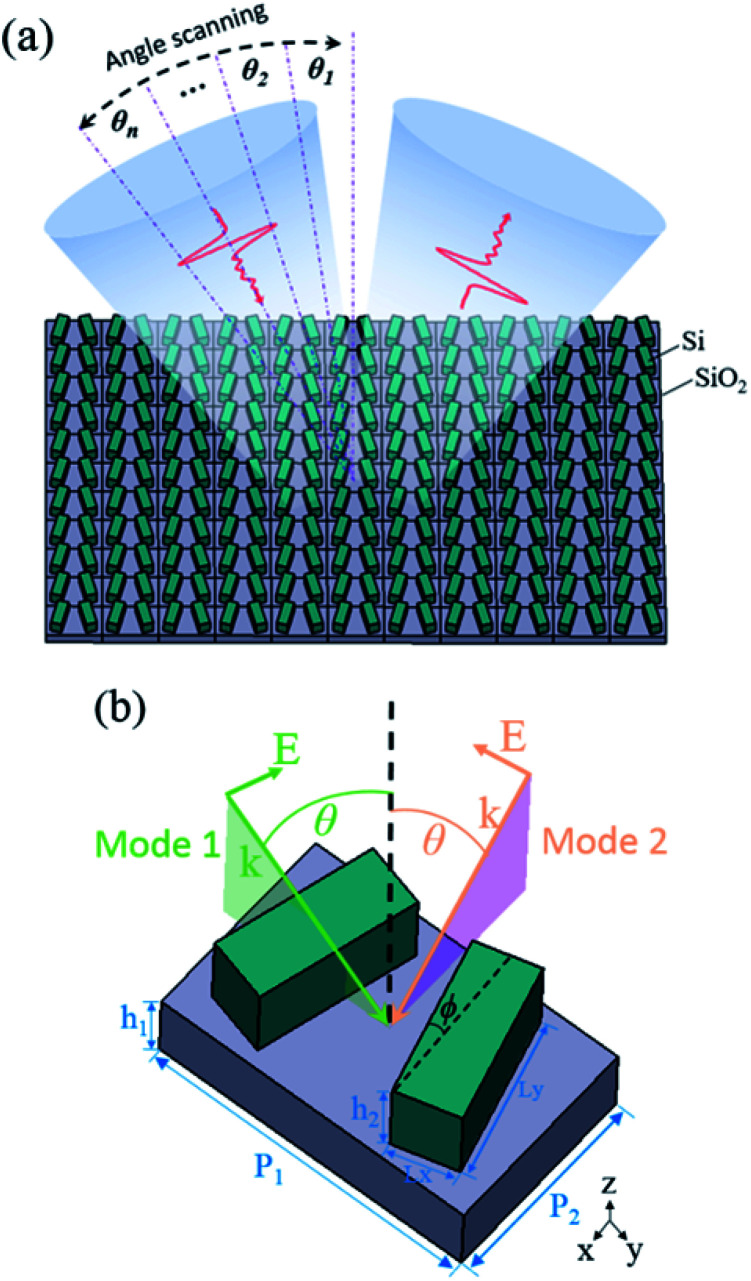
(a) Conceptual view of the all-dielectric metasurface sensor. Resonance peak will be shifted by continuous incident angle scanning, and eventually the reflection spectrum composed of a large number of resonances will cover the absorption peak of analytes in the THz band. (b) The structure of the unit cell, which consists of a zigzag array of line dipoles on a SiO_2_ substrate.

The conceptual view of the all-dielectric metasurface sensor is illustrated in Fig. 1(a). The metasurface consists of a zigzag array of tilted silicon bars on a SiO_2_ substrate. When the target material is attached to the sensor, the reflectance spectrum can be measured using a reflective THz-TDS system. In the reflective system, the reflection spectra represents the absolute amplitude of reflectance. By analyzing the amplitude variation of the reflection spectrum, the absorption of the analytes can be deduced. By varying the incident angle, the resonance peak will be shifted, and eventually the reflection spectrum composed of a large number of resonances will cover the absorption peak of analytes in the THz band, which is better for identifying absorption peaks of a trace of target sample than the sensors without the surface electric field enhancement.

**Fig. 2:**
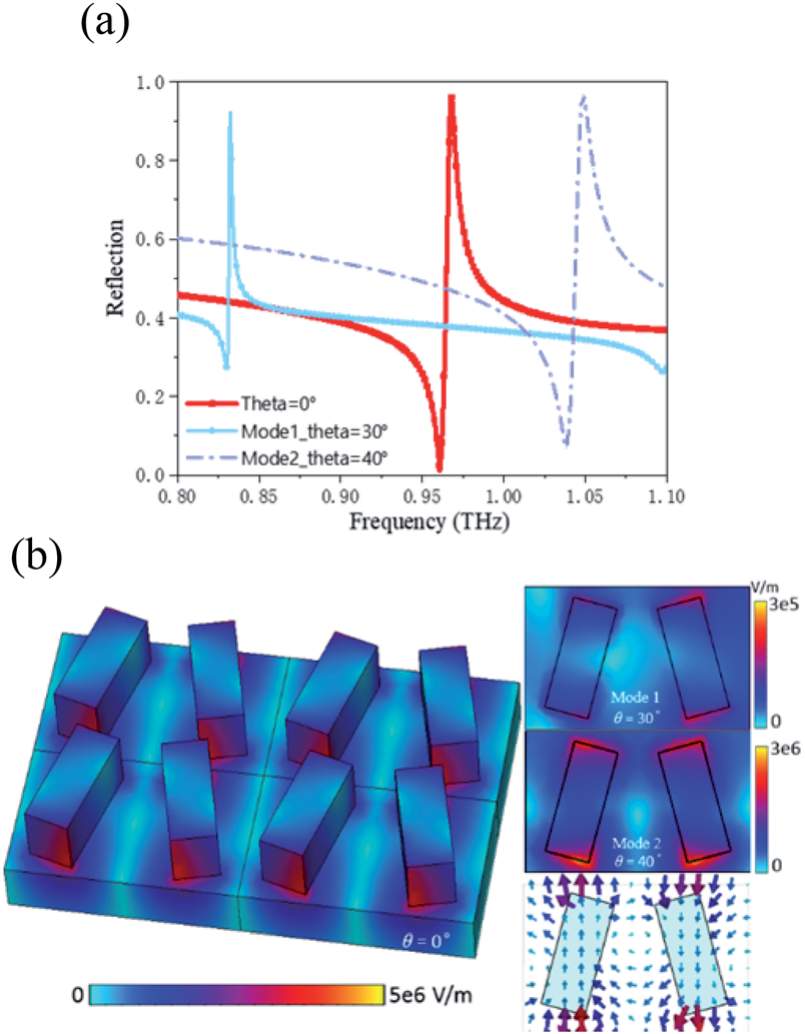
(a) Different reflection coefficients obtained at different incident angles in the two modes. (b) Absolute value distribution of the electric field and surface electric field direction in different modes.

In order to allow the reflectance spectrum to cover a wider band, the sensor works in two modes, which corresponds to incident angles of THz waves scanned in two different planes (*yz* and *xz*), as shown in Fig. 1(b). Hereafter, the *k* vector and the polarization of THz waves are in the *yz* plane in mode 1, whereas in the *xz* plane in mode 2. The metasurface resonance can be excited as long as the incident THz waves provide a nonzero electric field component along the *y* axis.^22^ Taking tyrosine recognition as an example, when THz waves irradiate metasurface vertically, the resonant frequency is 0.968 THz, close to the absorption peak of tyrosine. In mode 1, when the incident angle is scanned to 30°, the resonance frequency is 0.832 THz. In mode 2, when the incident angle is scanned to 40°, the resonance frequency is 1.05 THz, as shown in Fig. 2(a).

The performance of all-dielectric metasurface was investigated using commercial software CST with the frequency domain solver. The metasurface with unit cell boundary conditions in *x*- and *y*-directions are applied for calculating the reflection parameters. The substrate adopts low-loss SiO_2_ with a relative permittivity *ε*_r_ = 3.75 and loss tan *δ* = 4 × 10^−4^, and the tilted bar resonators are lossy silicon with a relative permittivity *ε*_r_ = 11.7 and loss tan *δ* = 2.6 × 10^−4^.^23^ Pairs of tilted bars on the SiO_2_ substrate can form a zigzag array. When the THz waves with polarization direction in the *y*-axis are incident on the metasurface, the resonance with high *Q* value will be excited, which originates from the physics of bound states in the continuum (BIC).^24,25^ A true BIC is a mathematical object with an infinite *Q* factor and vanishing resonance width, and it can exist only in ideal lossless infinite structures or for extreme values of parameters.^26,27^ By breaking the symmetrical structure of the unit cell, BIC is converted to quasi-BIC,^24^ and the finite *Q* value and resonant peak width can be realized, which is more suitable for its applications. By tilting the angle of the silicon bar, the simulation results show that the electric field on the surfaces of the two resonators is in opposite directions, forming an asymmetric dipole resonance. It can be seen from the electric field distribution shown in Fig. 2(b) that the electric field is bound at both ends of the bar resonator, which is beneficial for the interaction between the analytes attached to the metasurface and the THz waves, so as to detect the fingerprint spectrum of the analytes.

Since the scattered field from an electric dipole is intrinsically anisotropic in the plane of the metasurface, the electromagnetic interaction of the unit cell is also anisotropic.^28^ Furthermore, when the incident angle is tilted, the phase of the THz waves that incident on each unit cell in the metasurface will be different, and the effective impedance will be changed. As a result, when the incident angle is scanned in two different modes, the electromagnetic interaction will provide different responses, as shown in Fig. 2(a). According to the simulation results in Fig. 2(b), when the terahertz waves are incident vertically, the electric field intensity on the surface of the metasurface is the strongest, and the electric field intensity of mode 1 is lower than that of mode 2, which is caused by the different electric field components in different modes.

**Fig. 3:**
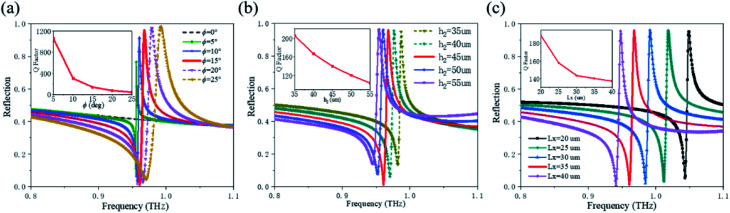
(a) The reflection coefficients for different orientation angles. Inset shows the trend of *Q* factor with variation of orientation angle. (b) The reflection coefficients for different resonator heights. Inset shows the trend of *Q* factor with different resonator heights. (c) The reflection coefficients for different resonator widths. Inset shows the trend of *Q* factor with different resonator widths.

The influence of the tilt angle of the silicon bar on the resonance was simulated, as shown in Fig. 3(a). The simulation results indicate that there is no sharp resonance excitation for the orientation angle *ϕ* of 0° because this structure supports a true optical BIC. In fact, such an ideal BIC is unstable against perturbations that break the in-plane symmetry,^24^ and with the increase of *ϕ*, it can transform into a quasi-BIC with a finite *Q* factor. For the Fano resonance, the *Q* factor can be estimated conveniently and quickly based on the difference of transmission peak and dip of the Fano resonance. Additionally, Fano fitting formula can also be used to fit the resonance peak and then calculate the *Q* factor, which is similar to the calculation result of previous method.^15^ In order to facilitate the calculation and comparison of the influences of structure parameters on *Q* factor, the corresponding *Q* factor of the metasurface have been calculated as *Q* = *f*/Δ*f*, where *f* is a resonant frequency and Δ*f* is defined as the difference of the frequency at transmission peak and dip.^15^ According to the simulation results in Fig. 3(a), it can be seen that when the orientation angle *ϕ* decreases, the *Q* value increases whereas the peak of resonance decreases. The main point of this paper is to use the envelope of resonant peak to detect the analytes. Therefore, the values of resonance peak are required to be as close as possible to 1. However, for refractive index sensing, the increase of *Q* factor is conducive to improving the sensing sensitivity. Consequently, a trade-off is made between the peak value and the *Q* factor, and finally the inclination angle is chosen to be 15°.

For the metasurface structure, the height and the shape of the resonator will also affect the resonance properties. Numerical simulation shows that the resonant frequency appears red shift and the *Q* factor decreases with the increase of the resonator height, as shown in Fig. 3(b). Meanwhile, as the value of *L*_*x*_ increases, the resonance also appears red shift and the *Q* factor decreases, as shown in Fig. 3(c). With the increase of the resonator height and width, the volume of the resonator increases and the ability of confining the electric field diminishes, which leads to the decrease of the *Q* factor. Among them, the increase of the resonator width brings about the increase of the electrical length of the resonator in the *y* direction, resulting in red shift of the resonance frequency. Since the target analyte of the sensor is tyrosine and there is a strong absorption peak near 0.97 THz, the resonant frequency of the metasurface should be close to the absorption peak of tyrosine when the THz waves are incident vertically. Finally, geometric dimension of the metasurface is set as *P*_1_ = 176 μm, *P*_2_ = 110 μm, *h*_1_ = 40 μm, *L*_*y*_ = 88 μm, *L*_*x*_ = 35 μm, *h*_2_ = 45 μm, and the *Q* factor is calculated to be 140. The thicknesses of SiO_2_ and silicon materials need to be customized, and the two sheets of materials can be combined into one sheet by the wafer bonding process. The technique of deep silicon etching is then used to lithograph the silicon structure on the surface of the wafer, which may require repetitive process experiments to ensure the fabrication quality of the sample.

**Fig. 4:**
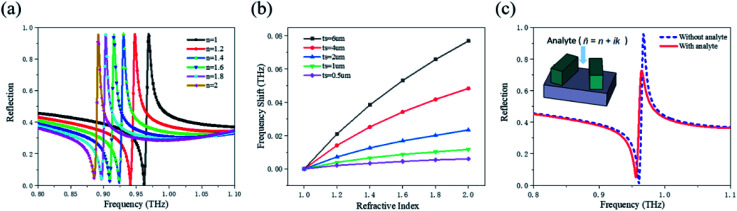
(a) The reflection coefficients for different refractive index of analyte. (b) Frequency shift *versus* the refractive index of the analyte located on the surface of metasurface for thickness changed from 0.5 μm to 6 μm. (c) Reflection simulation results for metasurface with and without tyrosine.

It is well known that the sharp resonance can be applied as an ultrasensitive sensor for refractive index sensing. Consequently, the sensing properties of optimized metasurface have further been investigated by numerical simulations. According to the simulation results, when the refractive index of the analyte increases from 1.0 to 2.0 in steps of 0.2, the resonant frequency is red shifted and total frequency shift of the resonance frequency is about 0.077 THz, as shown in Fig. 4(a). The sensitivities is 77 GHz/refractive index unit (RIU) and the standard sensitivities of resonances are obtained using the formula 
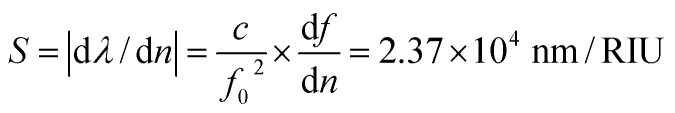
, here *c* is the speed of light in vacuum, *f*_0_ denotes the resonance frequency, and *n* represents the refractive index of the analyte. For the sensing application, FOM is usually applied to evaluate the performance of the ultrasensitive sensor. The FOM of this sensor can be calculated using the formula FOM = *S*/Δ*λ* = 11.1, which is much greater than that of metasurface sensor based on metal structure.^29,30^

For ultrasensitive metasurface sensors, the thickness of analyte also affects their sensing characteristics. Thus, the influence of the analyte thickness on the response of the resonance is investigated. Fig. 4(b) depicts the frequency shift of the resonator computed for different values of the thicknesses with refractive index of 2, where we observe a significant decrease in frequency shift with the decreasing analyte thickness.

In order to further analyze the influence of analyte on sensors, the reflectance spectrum of the metasurface with and without analyte has been numerically simulated, as shown in Fig. 4(c). The reflection peak decreases significantly with analyte attached to the metasurface. Additionally, when there are few analytes, the resonance frequency shift is negligible. Therefore, the metasurface is very suitable for detecting the fingerprint spectrum in the scanning mode.

## 3. Results and discussion

For most of the sensors, higher *Q* factor with tighter confined electromagnetic field usually means higher sensitivity,^21^ which is expected for refractive index sensing. However, the high *Q* factor implies the narrow linewidth of resonance, which is difficult to realize the specific sensing for analyte with broad absorption band. In this paper, the metasurface based on all-dielectric is proposed to make up for this lack in the THz band.

**Fig. 5:**
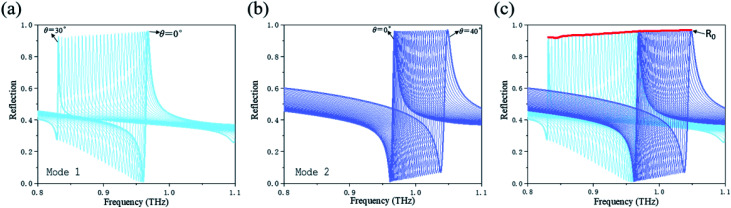
(a) Reflection spectra of the metasurface before analyte coating in mode 1, with scanning angular ranging from 0° to 30°. (b) Reflection spectra of the metasurface before analyte coating in mode 2, with scanning angular ranging from 0° to 40°. (c) The combined reflectance spectrum of mode 1 and mode 2, where the red curve (*R*_0_) represents the envelope of peak of the reflection curve.

Different from the metasurface sensor designed based on quasi-BIC theory in infrared band,^22,28^ in this paper, we use the more penetrating terahertz waves to irradiate the all-dielectric metasurface and then identify the absorption peak of the analytes. Compared with the traditional terahertz detection system, it can effectively promote the development of identifying trace chemical analytes by using terahertz waves. It has been proved above that the metasurface can realize resonance frequency shift with angle scanning in two modes. When the THz waves are incident vertically, the resonant frequency of the metasurface is close to the frequency of the tyrosine absorption peak. In mode 1, when the incident angles of THz waves are scanned from 0–30°, the resonance frequency shifts from 0.968 THz to 0.832 THz, as shown in Fig. 5(a). In addition, in mode 2, when the incident angles of THz waves are scanned from 0 to 40°, the resonance frequency shifts from 0.967 THz to 1.048 THz. By combining the reflectance spectra of the two modes, the resonance frequency can be scanned from 0.832 THz to 1.048 THz, which covers the absorption band of tyrosine. In order to retrieve the absorption spectrum of the analyte, the envelope of combined reflection peak is calculated, shown in Fig. 5(c).

In order to verify the specific sensing of the metasurface, tyrosine has been selected as the analyte, which plays an important role in the metabolism and growth of humans and animals.^31^ Tyrosine is a key precursor for neurotransmitters and hormone such as dopamine, norepinephrine and thyroxin in mammalian central nervous systems.^32,33^ Many analytical methods such as chemiluminescence detection, electrochemical sensor, spectroscopic analysis, and high performance liquid chromatography have been reported for the determination of tyrosine.^34–37^ Different from the existing methods, the metasurface sensor focuses on the identification of trace amounts tyrosine, and can be extended to identify other trace amounts analytes.

In order to obtain the optical constant of tyrosine in the THz band for the metasurface simulation, according to the method in ref. 38, it is necessary to prepare the pressed sample with the shape of disk for measurement by the THz-TDS. Considering that the adhesion of pure tyrosine is not good enough and the pressed sample is easy to be crushed, it is necessary to use the polyethylene powder to press together, which is nearly transparent in the THz range and can be pressed into a small disk. The samples consisted of a mixture of 62 mg tyrosine and 146 mg polyethylene powder, which were pressed with a force of 400 kg into a disk with a diameter of about 13 mm and a thickness of 1.32 mm. By measuring the sample in the THz-TDS system, the transmission spectrum of the tyrosine can be obtained, and then the optical parameters of pure tyrosine can be calculated. The data processing refers to the formula in ref. 38–40. First, the complex refractive index of the samples can be expressed as *N* = *n* + i*k*, and the transmitted field *T*(*ω*) = *E*_s_(*ω*)/*E*_r_(*ω*)can be expressed as^39,40^1
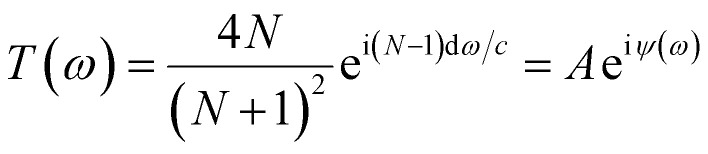
where *ω* and *A* are the frequency and amplitude of the THz wave, respectively, *ψ*(*ω*) is the phase difference between the reference waveforms and sample, *c* is the speed of light in vacuum and *d* is the thickness of the sample. Then, we can obtain the refractive index *n*(*ω*) and the absorption coefficient *a*(*ω*) of the mixture:^38^2
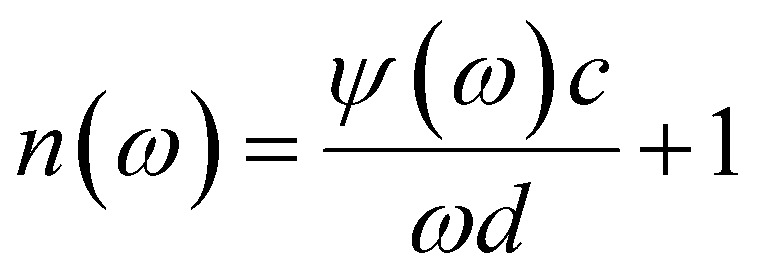
3
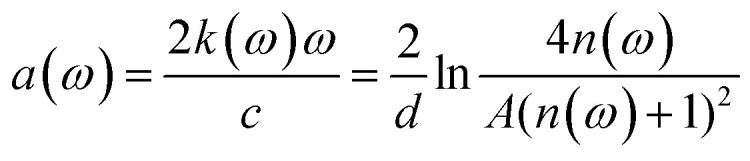


**Fig. 6:**
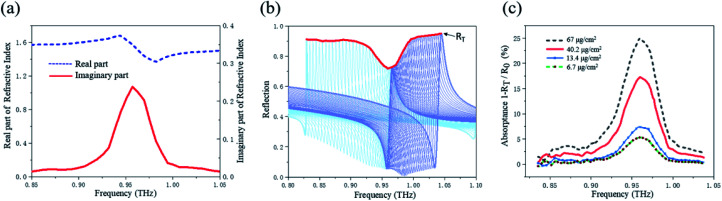
(a) Real part (blue dash line) and imaginary part (red solid line) of tyrosine obtained by measuring the sample with the shape of disk. (b) The simulation results of the combined reflection spectrum of the two modes after analytes coating, where the red curve (*R*_T_) represents the envelope of reflection peak. (c) The simulation results of absorption spectra of tyrosine film with different thicknesses.

In order to obtain the parameters of the pure sample, the following treatment is required:4
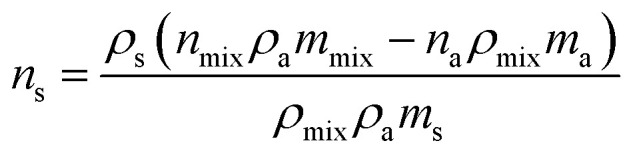
5
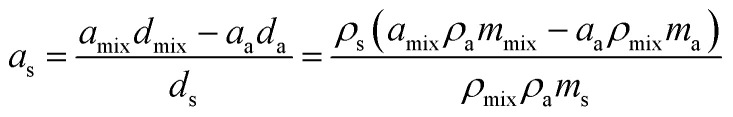
where *n*_s_ and *a*_s_ are refractive index and the absorption coefficient of pure sample, respectively, *ρ*_s_, *ρ*_a_ and *ρ*_mix_ represent the density of the pure sample, the polyethylene, and the mixture, *m*_s_, *m*_a_ and *m*_mix_ represent the mass of the pure sample, the polyethylene, and the mixture, respectively. The calculation results are shown in Fig. 6(a).

The refractive index and extinction coefficient of tyrosine are substituted into the simulation model to calculate the reflection spectrum with the incident angle scanning in two modes, as shown in Fig. 6(b), where the red curve (*R*_T_) represents the envelope of the reflection peak.

The absorption spectrum of tyrosine can be obtained by calculating the envelope of reflection peak before (*R*_0_) and after (*R*_T_) the attached analytes. After the end of angle scanning, the overlapping reflection curves of peaks are removed at first, and the maximum values of each reflection curve are taken to further form discrete and discontinuous groups of extreme points. Then, the envelope curve is obtained by using the linear interpolation operation to interpolate the extreme points. Finally, the absorption spectrum of the analytes can be calculated by the use of the relation (1 − *R*_T_/*R*_0_) × 100%. The absorption spectrum shown in Fig. 6(c) is approximately the same as that of the pure tyrosine shown in Fig. 6(a), which indicates that the specific sensor based on all-dielectric metasurface can accurately identify tyrosine. In order to further analyze the sensing capability for trace amounts analytes, the absorption spectra are presented in Fig. 6(c) with various surface densities (thicknesses as 0.5, 0.3, 0.1, 0.05 μm) of tyrosine.

Considering that the signal to noise ratio for an actual THz-TDS system is usually higher than 1000, the calculated result will not highlight the absorption peak of tyrosine when the thickness of the surface analyte in the simulation is less than 0.05 μm. Therefore, by substituting the density value of tyrosine (1.34 g cm^−3^) into the calculation, the final detection limit of the specific THz sensor for tyrosine is set as 6.7 μg cm^−2^. In the THz-TDS system, the spot diameter of THz waves can be adjusted to 3 mm by lens focusing. The THz waves spot can be completely covered by a sensor with a surface size of 1 cm × 1 cm, thus the absorption peak in THz band can be obtained by using only 6.7 μg tyrosine.

**Fig. 7:**
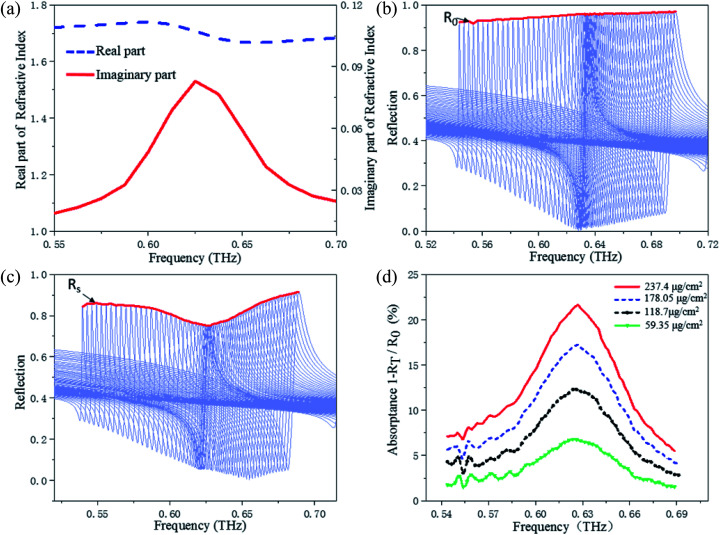
(a) Real part (blue dash line) and imaginary part (red solid line) of santonin obtained by measuring the sample with the shape of disk. (b) The simulation results of the combined reflectance spectrum of mode 1 and mode 2, where the red curve represents the envelope of peak of the reflection curve. (c) The simulation results of the combined reflectance spectrum of two modes after analyte coating, where the red curve (*R*_s_) represents the envelope of reflection peak. (d) The simulation results of the absorption spectra of santonin film with different thicknesses.

In order to further verify the universal adaptability of the specific sensor, santonin was selected as another target analyte. Similar to the preparation of tyrosine, the santonin with the shape of disk were prepared so that optical parameters of santonin in THz band could be obtained for the metasurface simulation. By measuring the santonin with a mass of about 381 mg and a thickness of 2.46 mm in the THz-TDS system, the transmission spectrum can be obtained, and then the optical parameters of santonin can be calculated by referring to the method of calculating tyrosine, as shown in Fig. 7(a). In order to use the same principle of the specific sensor to identify trace amounts of santonin, the size of the resonant unit can be proportionally adjusted, and the optimized size is set as *P*_1_ = 270 μm, *P*_2_ = 169 μm, *h*_1_ = 60 μm, *L*_*y*_ = 135 μm, *L*_*x*_ = 53 μm, *h*_2_ = 70 μm.

Similar to the tyrosine recognition, when THz waves are incident vertically, the resonant frequency of the metasurface is close to the frequency of the santonin absorption peak at 0.625 THz. In mode 1, when the incident angles are scanned from 0 to 30°, the resonance frequency shifts from 0.633 THz to 0.544 THz. In mode 2, when the incident angles are scanned from 0 to 45°, the resonance frequency shifts from 0.633 THz to 0.697 THz. By combining the reflectance spectra of the two modes, the reflection spectrum can cover the absorption spectrum of santonin. The envelope of combined reflection peak is calculated and shown in Fig. 7(b). After substituting the refractive index and extinction coefficient of santonin into the model for calculation, the reflectance spectrum covered with santonin can be obtained, as shown in Fig. 7(c). It can be seen from the calculation results that the absorption spectrum exhibits a strong absorption in 0.625 THz, which is consistent with the reference absorption peak. Due to the absorption strength of santonin in the THz band is less than tyrosine, the coverage thicknesses of the santonin are set as 2 μm, 1.5 μm, 1 μm and 0.5 μm, respectively, and the absorption intensity decreases with the decrease of thicknesses, as shown in Fig. 7(d). Considering the signal to noise ratio of the actual THz-TDS system, the final detection limit of the santonin is set as 59.35 μg cm^−2^.

## 4. Conclusions

In summary, a high sensitivity specific sensor based on all-dielectric metasurfaces with frequency scanning in the THz range was designed. The unit structure of the metasurface consists of a zigzag array of tilted silicon bars on a SiO_2_ substrate, which can excite a high *Q* resonance based on quasi-BIC. By simulating and analyzing the influence of different parameters on the resonance, the geometric parameters of the resonator have been optimized to achieve a tradeoff between *Q* factor, tunability range and the center frequency. When the incident angles of THz waves are scanned in two different polarization modes, the resonant frequencies will be red shifted and blue shifted, respectively, which eventually covers a wide reflection spectrum. After analyzing the refractive index sensing characteristics of the metasurface and the influence of thickness on the sensor, it is proved that the absorption peak of tyrosine at 0.97 THz and the absorption peak of santonin at 0.625 THz can be successfully recognized by the metasurface sensor. Additionally, the absorption spectra of analyte films with different thickness were calculated and indicated that the detection limit of the tyrosine and the santonin are 6.7 μg cm^−2^ and 59.35 μg cm^−2^, respectively. The high sensitivity specific sensors based on the all-dielectric metasurfaces with resonant frequency scanning exhibit significant potential for identification of fingerprint spectrum.

## Conflicts of interest

There are no conflicts to declare.

## Supplementary Material
